# Comparative Insights into Cutaneous, Mucosal, and Vulvovaginal Melanomas: Biology, Targeted Therapies, and Survival with a Focus on Immune Checkpoint Inhibitors

**DOI:** 10.3390/jpm15110551

**Published:** 2025-11-12

**Authors:** Danielle Christmas, Christina Pappa, Catherine Howell, Mohammad Daas, Keith Howell, Sunanda Dhar, Binamra Sigdel, Sanjiv Manek, Moiad Alazzam

**Affiliations:** 1 Department of Gynaecological Oncology, Oxford University Hospitals, NHS Foundation Trust, Oxford OX3 9LE, UK; danielle.christmas@ouh.nhs.uk (D.C.); christinapappa4@gmail.com (C.P.); binamras@gmail.com (B.S.); 2 GynaeFellow Research Collaborative, Old Road, Oxford OX3 7LE, UKmo.daas1996@gmail.com (M.D.); 3 Calderdale and Huddersfield, NHS Foundation Trust, Women’s Health, Halifax HX3 0PW, UK; 4 Department of Obstetrics and Gynaecology, Basildon and Thurrock University Hospital, Basildon SS16 5NL, UK; 5 Leeds Cancer Centre, St James’s University Hospital, Leeds Teaching Hospitals, Beckett Street, Leeds LS9 7TF, UK; keith.howell@nhs.net; 6 Department of Cellular Pathology, Oxford University Hospitals, NHS Foundation Trust, Oxford OX3 9LE, UK; sunanda.dhar@ouh.nhs.uk (S.D.); sanjiv.manek@ouh.nhs.uk (S.M.)

**Keywords:** cutaneous melanoma, mucosal melanoma, vulvovaginal melanoma, immunotherapy, targeted therapy, *BRAF* mutation, tumour microenvironment

## Abstract

**Background/Objectives**: Melanoma is a malignant tumour of melanocytes. Cutaneous melanoma accounts for the vast majority of cases and has benefitted from advances in targeted and immune checkpoint inhibitor therapies, leading to substantial improvements in survival. In contrast, mucosal and vulvovaginal melanomas are rare, aggressive subtypes with distinct molecular and immune profiles and poor prognoses. This review synthesises evidence comparing cutaneous, mucosal, and vulvovaginal melanoma, with emphasis on biology, treatment, and outcomes **Methods**: A narrative comparative review was undertaken, examining the published literature on the epidemiology, molecular and immune characteristics, and treatment outcomes of cutaneous, mucosal, and vulvovaginal melanoma, including systemic therapies and surgical approaches. **Results**: Cutaneous melanoma demonstrates high tumour mutational burden and frequent BRAF and *NRAS* mutations, underpinning the success of targeted therapy and immunotherapy. Mucosal and vulvovaginal melanomas exhibit lower mutational burden, distinct mutation patterns, and reduced immunogenicity, correlating with poorer treatment responses. Surgery remains the mainstay of management, though optimal margins in vulvovaginal melanoma are unclear. Recurrence rates are high, and five-year survival remains poor. Evidence for systemic therapy is limited to small retrospective cohorts and subgroup analyses, showing lower response and survival rates compared with cutaneous melanoma. Chemotherapy has minimal benefit. **Conclusions**: Mucosal and vulvovaginal melanomas are biologically and clinically distinct from cutaneous melanoma and continue to have poor survival outcomes. Their rarity restricts high-quality evidence, highlighting the need for collaborative, innovative research to inform effective treatment strategies.

## 1. Introduction

Melanoma is a malignant neoplasm arising from melanocytes. These are pigment cells located in the epidermis and within mucosal membranes of the upper airway, genitourinary tract, and gastrointestinal tract. The most common melanoma subtype, cutaneous melanoma, arises from melanocytes within the epidermis, whereas the significantly rarer mucosal melanomas arise from melanocytes in mucosal membranes. Traditionally, these pathologies have been seen to be similar and findings from one sub-type have often been extrapolated. Recent evidence suggests that although arising from a similar cell origin, melanomas are a highly heterogenous group and demonstrate significant differences in their underlying pathogenesis, immune microenvironment, genetic mutations, clinical behaviour and subsequent response to treatment. The more common cutaneous melanoma has seen improved survival over the past decade as a result of significant advances in the understanding of tumour biology and the development of new targeted and immune checkpoint inhibitors (ICIs). Our understanding of immune checkpoint pathways and the *BRAF*/*CKIT* genetic mutations which underpin the pathogenesis of many cancers has revolutionised cancer treatment. ICIs targeting cytotoxic T-lymphocyte-associated protein 4 (CTLA-4) and programmed cell death protein 1 (PD-1) or its ligand (PD-L1) inhibit negative immune regulation, thereby enhancing anti-cancer immune activity. In contrast, partly due to their rarity, understanding of the distinct features of mucosal and vulvovaginal melanomas has progressed at a slower rate and the evidence used to guide treatment of these tumours is extremely limited, with only small retrospective studies and case series or largely extrapolated from cutaneous melanoma trials. This review synthesises current evidence to compare cutaneous melanoma, mucosal melanoma and particularly the subset of vulvovaginal melanoma as three distinct subtypes. The molecular and mutational changes, immune profile and how these changes translate to the efficacy of treatment and patient survival are explored to identify key areas for future progress.

## 2. Materials and Methods

Given the broad scope of this review—encompassing epidemiology, tumour biology, therapeutic options, and survival outcomes across three disease pathologies—a narrative design was chosen. A literature search was undertaken in MEDLINE (PubMed), Embase, and ClinicalTrials.gov to identify studies on cutaneous, mucosal, and vulvovaginal melanomas published up to 30 June 2025. The search strategy combined relevant keywords and Medical Subject Headings (MeSH) to perform several targeted literature reviews tailored to the specific focus of each component of the review. These keywords included: cutaneous melanoma, mucosal melanoma, vulvovaginal melanoma, *BRAF* mutation, NRAS mutation, *KIT* mutation, genetic mutation, tumour microenvironment, ICIs, PD-1 inhibitors, CTLA-4 inhibitors, targeted therapy, *BRAF*/*MEK* inhibitors, systemic therapy, adjuvant therapy, surgical management, recurrence, and survival outcomes. For example, keywords including ‘adjuvant treatment’ OR ‘immune checkpoint inhibitors’ OR ‘systemic treatment’ AND ‘vulvovaginal melanoma’ were used when performing the literature search for ICI use in vulvovaginal melanoma. This allowed for comprehensive and targeted identification and synthesis of studies, however, it also limits the ability to present a single flow of screened and excluded studies. Systematic reviews, randomised controlled trials, cohort and observational trials (both prospective and retrospective) were included. Case series and case reports were excluded. Due to logistical considerations, only English-language publications were included, which was a limitation of the study given the higher incidence of mucosal melanoma in Asian populations. Additionally, given the narrative nature of this review, a formal quality appraisal of each study was not undertaken. Additional relevant articles were identified by screening the reference lists of relevant studies. Meta-analysis of Observational Studies in Epidemiology—A Proposal for Reporting was used, where relevant, for included observational studies.

## 3. Discussion

### 3.1. Epidemiology, Presentation, Risk Factor

#### 3.1.1. Cutaneous Melanoma

Cutaneous melanomas account for the majority of melanoma diagnoses (>90%) in white populations, with a steady rise in incidence worldwide since the 1950s. This has been attributed to greater exposure to ultraviolet radiation (UVR), both from natural and artificial sources [1]. Reported global incidence of melanoma in 2020 was 3.4 per 100,000 [2], with the highest incidence rates observed in Australia and New Zealand at 42 per 100,000 person-years in males and 31 per 100,000 person-years in females [3]. Cutaneous melanoma arises from the progressive accumulation of genetic mutations, largely driven by the oncogenic effects of UVR, which disrupt normal cell proliferation, differentiation, and death. Additional interactions between inherited germline genetic modifiers and phenotypic risk factors (e.g., lighter skin tone, sun sensitivity, naevus count or type) also contribute. Accordingly, risk factors for cutaneous melanoma include UVR exposure and non-modifiable factors such as male sex, high naevus count, atypical naevi, first-degree relative with melanoma, previous melanoma, and immunosuppression [1]. Melanoma tumours are classified into nine subtypes, a system originally developed by the World Health Organization (WHO), with each subtype characterised by distinct epidemiological, clinical, histopathological, and genomic features. The two most common forms of cutaneous melanoma are low cumulative sun damage melanoma (superficial spreading melanoma) and high cumulative sun damage melanoma (lentigo maligna melanoma). Superficial spreading melanoma usually arises in sun-exposed skin with minimal solar elastosis on the trunk or back of younger adults. In contrast, lentigo maligna melanoma typically occurs on the head and neck of older patients and is associated with marked solar elastosis and chronic sun exposure [4]. Fortunately, with contributions from health education and awareness campaigns, cutaneous melanoma is predominantly diagnosed at early stages, with 77% of patients having localised disease at diagnosis [5].

#### 3.1.2. Mucosal Melanoma

Mucosal melanoma is a rare and aggressive subtype, accounting for less than 2% of all melanomas in white populations and up to 23% in Chinese populations [6]. Unlike cutaneous melanoma, its incidence appears relatively stable. Mucosal melanomas can arise from any mucosal membrane and most commonly occur in the head and neck, anorectal, and vulvovaginal regions. They are more frequent in females, largely due to the occurrence of vulvovaginal melanomas, which represent the most common subtype in women, while head and neck tumours are the most common subtype in men [7,8]. Mucosal melanomas are usually diagnosed later in life, with a median age of 70 years [8]. Their risk factors remain poorly understood. Unlike cutaneous melanoma, UVR does not appear to be a strong causative factor, and many sites in which mucosal melanoma develops are not routinely exposed to UVR [9]. Mucosal melanoma is often diagnosed at a later stage, with two-thirds of patients presenting with regional, distant, or nodal metastasis at the time of diagnosis. This may be partly due to a lack of specific symptoms or signs and the wide variation in clinical presentation, depending on the site of origin, which can delay diagnosis. The sites are often more occult than those of cutaneous melanoma. These factors likely contribute to the poor prognosis, with overall five-year survival rates of only 25% despite optimal surgical resection [10].

#### 3.1.3. Vulvovaginal Melanoma

Vulvovaginal melanomas have traditionally been grouped with mucosal melanomas, but recent evidence examining their molecular and genetic characteristics shows that melanomas of the female genital tract differ from both other mucosal and cutaneous melanomas [11]. Vulvovaginal melanomas are extremely rare, accounting for 1% of all malignant melanomas in women, 5.3% of vulval malignancies, and 5.5% of vaginal malignancies [11]. The reported incidence is about 1.74 cases per million person-years [12]. The median age at diagnosis is 68 years for vulval melanoma and 71 years for vaginal melanoma. Risk factors remain poorly understood but include advanced age and white ethnicity [13]. Diagnosis often occurs at an advanced stage, with regional or distant metastatic disease present in 45.6% of vaginal melanoma patients and 31.6% of vulval melanoma patients. In both groups, about one-third also have nodal disease at diagnosis [11]. Prognosis is worse compared with cutaneous melanoma, with reported five-year overall survival rates of 37% for vulval melanoma and 13–32% for vaginal melanoma [14]. Women with vaginal melanomas appear to have the poorest prognosis, with survival outcomes consistently worse than those of vulval melanoma patients across all stages, and a reported median overall survival of 16 months [11]. Refer to Table 1.

### 3.2. Biology and Immune Profile

#### 3.2.1. Cutaneous Melanoma

The progression of melanoma results from interactions between environmental, genetic, and host factors. UVR induces DNA damage, with a higher number of mutations observed in cutaneous melanoma—particularly desmoplastic subtypes—compared with the low tumour mutational burden seen in mucosal melanomas. Somatic mutations also contribute to melanoma development and progression, with alterations in the mitogen-activated protein kinase (MAPK) signalling cascade representing a key pathogenic pathway present in about 90% of cutaneous melanomas [15]. These mutations may occur in receptors (e.g., *KITKIT*), effectors (*BRAF*, *NRAS*), or inhibitors (*NF1*, *RASA2*, *CDKN2A*, *SPRED1*) of the pathway, leading to proliferation of transformed melanocytes, abnormal differentiation, and persistent survival with impaired apoptosis of tumour cells. The reported frequencies of mutations in cutaneous melanoma are 37–60% in *BRAF* [15,16], 13–25% in *NRAS*, 12% in *NF1*, 6–7% in *MAP2K1*, and 2–8% in *KIT* [4,16]. Up to 78% of melanomas also carry a *TERT* promoter mutation, which increases telomerase activity and allows cells to maintain telomeric elongation, resulting in prolonged survival and abnormal proliferation. This mutation is often seen with *BRAF* and *NRAS* mutations, as the increased telomerase expression stabilises the mutated *BRAF* and *NRAS* genome [17]. Accordingly, *TERT* positivity is associated with poorer patient survival. The tumour mechanisms that evade normal immune responses are increasingly understood in cutaneous melanoma and have provided meaningful targets for immunotherapy. Tumour-infiltrating lymphocytes, Natural Killer (NK) cells, and CD8+ T cells can initially control melanoma progression through antigen-specific cytotoxic mechanisms that destroy tumour cells. However, tumour cells promote an immunosuppressive and pro-tumorigenic environment through the release of interferons, interleukins, and colony-stimulating factors [4]. In malignant cells, interferons drive excessive activation of the JAK1/2 and STAT kinase pathways, which stimulate the production of PD-L1 and PD-L2 ligands. These ligands are transported to the cell membrane and presented to T lymphocytes, which express PD-1checkpoints, thereby protecting the tumour cell from immune-mediated destruction [13]. The tumour microenvironment also influences the response to immunotherapy, whereby immunologically ‘hot’ tumours display high tumour mutational burden, greater numbers of tumour-infiltrating lymphocytes, and tumour-secreted interferon, and are therefore more responsive to immunotherapy. Such features are commonly seen in cutaneous melanoma, underpinning the effectiveness of immunotherapy in these tumours [4].

#### 3.2.2. Mucosal Melanoma

Genetic alterations in mucosal melanoma are distinct from those in cutaneous melanoma. Compared with cutaneous melanoma, mucosal melanomas show lower rates of somatic mutations but greater genomic instability. Mutations in *BRAF* and *NRAS* are less common (up to 20% of patients) and, when present, usually involve non-canonical non-V600 mutations. Mutation frequencies also vary across specific anatomical sites, with head and neck melanomas more often showing mutations in *NRAS* and *TERT* promoters, and genitourinary or anorectal melanomas showing *KITKIT* mutations [18,19].

**Figure 1:**
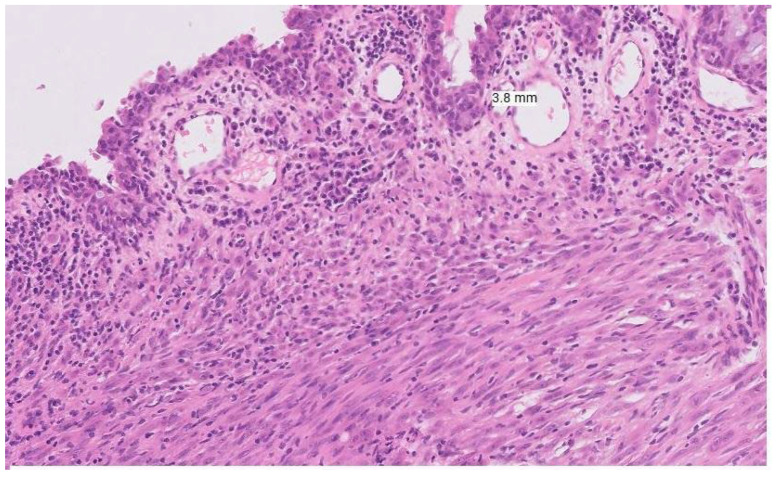
Histopathological image of mucosal melanoma showing atypical melanocytes infiltrating the submucosa.

Mucosal melanomas are less immunogenic and generally have a lower tumour mutational burden, particularly those arising in the anorectal and urogenital tracts compared with conjunctival or nasal sites [16,20]. This site-specific variation may partly reflect the mutagenic role of UVR, which influences the distinct mutational signatures of mucosal melanomas in facial sites compared with those in the lower body [20]. The tumour immune microenvironment in mucosal melanomas also shows reduced immune cell infiltration and a weaker interferon-gamma signature compared with cutaneous melanoma—a difference that is especially marked in urogenital tumours compared with those of the head and neck [21] (See Figure 1). The above distinct genetic alterations and lower immunogenicity with low tumour mutational burden contribute to reduced efficacy of systemic therapies and ICIs. These factors combined with delayed and late diagnosis, significant heterogeneity within the mucosal melanoma group and challenges of surgical resection, contribute to the aggressive nature of this tumour group.

#### 3.2.3. Vulvovaginal Melanoma

**Figure 2:**
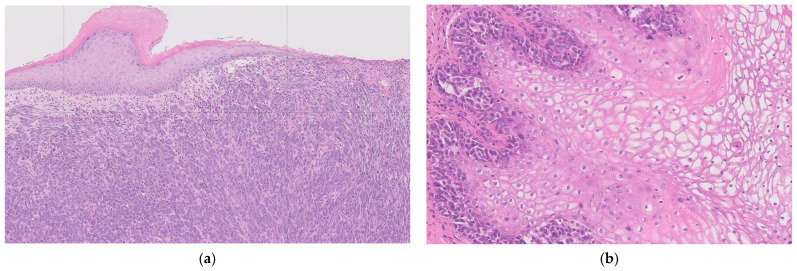
Histopathological Features for (**a**) Vulval Melanoma; Sections show a pigmented melanocytic lesion with evidence of junctional activity, marked cytological atypia, and invasion into the deep dermis. Growth is seen in both radial and vertical phases, with a brisk mitotic rate and perineural invasion. Melanin pigment is present within tumour cells. (**b**) Vaginal Melanoma; The tumour shows a polypoid architecture, often pigmented, with satellite nodules. Microscopy demonstrates solid, nested, and trabecular patterns, with a radial growth phase and brisk mitotic activity. Melanin is frequently present. Immunohistochemistry highlights S100 as the most sensitive marker.

Emerging evidence highlights the distinct molecular profile and tumour microenvironment seen in vulvovaginal melanoma, with variation evident even between vulval and vaginal sites. *KITKIT* mutations are more common in vulval melanomas (22–31% of cases) compared with vaginal melanomas (8%) [22,23]. Conversely, *NRAS* mutations are uncommon in vulval melanomas (10.2%) and appear to have a comparatively higher incidence in vaginal melanomas [22,23]. *BRAF* mutations are significantly less common in vulvovaginal melanomas, and *TERT* mutations may be entirely absent. Other frequently mutated genes include ATRX, SF3B1, B2M, NF1, and TP53 [23]. In terms of immunogenicity, vulvovaginal melanomas demonstrate lower rates of PD-L1 positivity (18%) compared with cutaneous melanomas (29.5%) and overall reduced expression of immune checkpoint genes. They also have a lower tumour mutational burden, in contrast to 46.9% of cutaneous melanomas, which display a high tumour mutational burden [23]. RNA deconvolution analysis of the tumour microenvironment further demonstrates reduced adaptive immune responses and decreased immunogenicity, with lower infiltration of immune-promoting macrophages, effector CD8+ T cells, and CD4+ T cells [23]. These distinct mutational and immune characteristics highlight vulvovaginal melanoma as a unique subclass of mucosal melanoma and may help explain its differential responses to systemic melanoma therapies (See Figure 2).

### 3.3. Treatment

#### 3.3.1. Surgery in Resectable Disease

In all melanoma types, when the disease is resectable, the standard of care is surgical resection of the primary lesion and, where possible, resectable metastatic disease. For cutaneous melanoma, this involves wide local excision at the primary site with a 1–2 cm radial surgical margin, depending on tumour thickness. Larger margins are not recommended by governing bodies in Europe, the United States, the United Kingdom, or Australia [24]. Non-surgical approaches, including topical imiquimod and radiotherapy, have been shown to be inferior to surgical excision [24]. As in cutaneous melanoma, surgery remains the mainstay of primary treatment for mucosal melanomas, including vulvovaginal subtypes however this conclusion is notably based only on retrospective observational studies as best level of evidence. This evidence suggests that primary surgery achieving negative margins provides the best survival outcomes [25,26]. Consensus guidelines and evidence to define the optimal surgical margin are lacking in vulvovaginal melanoma and therefore remain unclear. Notably, increased radicality of surgery has not been shown to improve survival outcomes compared with wide local excision with negative margins in retrospective analyses [27,28]. Vulvovaginal melanomas present unique surgical challenges due to their proximity to critical structures such as the urethra, bladder, anus, and rectum; thus, achieving negative margins alone may warrant more radical surgery or exenterative procedures [29].

#### 3.3.2. Lymph Node Assessment

Lymph node assessment is a key part of managing newly diagnosed cutaneous melanoma for selected patients, including those with T2 or higher tumours or high-risk T1 melanoma. In this group, sentinel lymph node assessment is now widely accepted for patients with clinically negative nodes and should be performed at the time of the initial excisional procedure. Historically, patients with a positive sentinel lymph node underwent completion lymph node dissection; however, this is no longer routinely recommended, as no survival benefit has been demonstrated, even in trials conducted before the introduction of more effective systemic therapies [30]. In vulvovaginal melanoma, there is a paucity of high-quality evidence to guide decision-making regarding lymph node assessment and the optimal modality for achieving this. Traditionally, the standard treatment for vulval melanoma has been full inguinofemoral lymphadenectomy, but this is associated with significant morbidity, including wound complications, infection, and lymphoedema. Large population-based retrospective analyses have demonstrated that lymph node status is the strongest predictor of overall survival [11], highlighting the importance of determining nodal disease in patients with vulvovaginal melanoma, particularly in the era of potentially effective adjuvant therapies.

In non-melanoma vulval cancer, sentinel node biopsy has replaced complete inguinofemoral lymphadenectomy for unifocal tumours smaller than 4 cm without suspicious nodal disease on imaging or examination [31]. However, there is very limited evidence to guide similar practice in vulvovaginal melanoma. A recent systematic review identified six retrospective studies assessing the use of sentinel node biopsy in vulval melanoma across 48 patients using either radioactive or blue dye methods. Of 32 patients with negative sentinel nodes, only two experienced groin recurrence [32]. These findings are limited by the small number of patients and the absence of prospective trials, meaning that sentinel node biopsy is not yet an established practice in vulvovaginal melanoma.

#### 3.3.3. Adjuvant Treatment Using Immune Checkpoint Inhibitors

**Figure 3:**
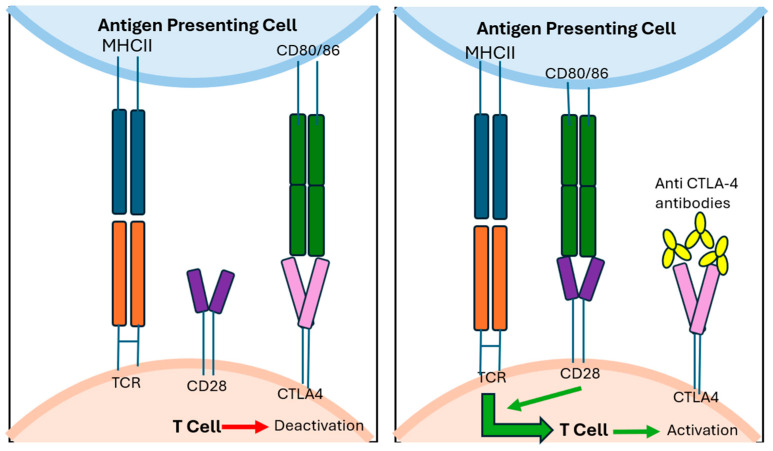
CD80/86 binds to CTLA4 inhibiting TCR activation through competition inhibition of CD28 stimulatory signalling. Anti-CTLA 4 antibodies prevent CD80/86 binding to CTLA 4 thereby enabling binding to CD28 and activation of stimulatory pathways.

**Figure 4:**
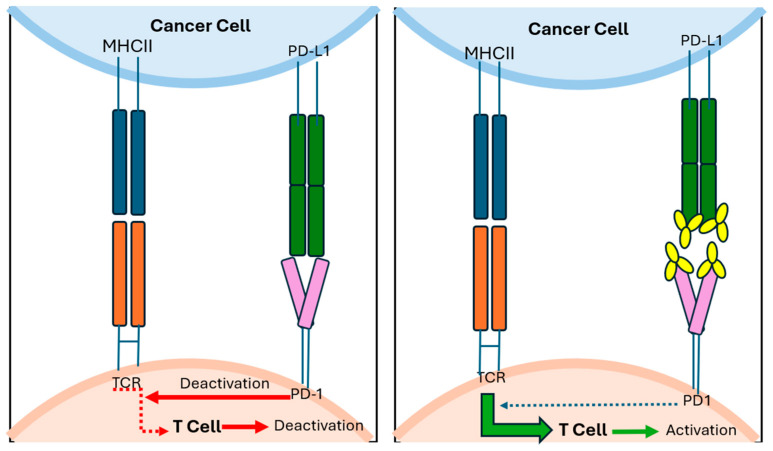
Tumour cells may express PD-L1 which can bind to the inhibitor immune checkpoint PD-1 on T cells causing an inhibition of T cell activation. Anti-PD1/PD-L1 antibodies prevent this binding thus reducing negative inhibition of T cell activation.

Despite optimal resection, recurrence is common across all melanoma subtypes. However, adjuvant therapy has been best established in cutaneous melanoma, more so than in mucosal and vulvovaginal melanomas. For patients with high-risk resectable cutaneous melanoma (stage IIB or higher), adjuvant treatment with checkpoint inhibitor immunotherapy (mechanisms demonstrated in Figure 3 and Figure 4) or *BRAF*/*MEK* targeted therapy is now the standard of care.

Adjuvant anti-PD-1 therapy (nivolumab or pembrolizumab) has demonstrated a significant recurrence-free survival benefit over both ipilimumab (CTLA-4 inhibitor) and placebo in resected stage III–IV melanoma across multiple prospective, randomised phase III trials [33,34]. These trials also showed that nivolumab and pembrolizumab were more tolerable than ipilimumab. Further trials, such as CheckMate 915, investigated whether outcomes in stage IIIB–IV melanoma could be improved by adding a low-dose regimen of ipilimumab to nivolumab, compared with nivolumab monotherapy. No significant recurrence-free or overall survival benefit was observed at two years, but the combination group experienced higher rates of treatment-related adverse events [35]. Thus, current evidence strongly supports a monotherapy approach with the ICIs nivolumab or pembrolizumab in the adjuvant treatment of advanced cutaneous melanoma. The effectiveness of immunotherapy in cutaneous melanoma reflects the tumour microenvironment and immunological changes that underpin its pathogenesis.

The efficacy of ICIs in the adjuvant setting is less well established in both mucosal and vulvovaginal melanoma, and there is currently no universally accepted standard for adjuvant treatment (included studies summarised in Table 2). Limited evidence suggests that ICIs are less effective in mucosal melanoma than in cutaneous melanoma. The SALVO trial assessed a ‘flip dose’ regimen of ipilimumab and nivolumab for adjuvant treatment of mucosal melanomas. Participants had undergone R0/R1 resection with no prior systemic therapy and received ipilimumab (1 mg/kg every three weeks for four cycles) and nivolumab (3 mg/kg every three weeks for four cycles), followed by nivolumab 480 mg every four weeks for 11 cycles to complete one year of adjuvant therapy. This trial reported recurrence-free survival rates of 50% and 37% at one and two years, respectively, with overall survival rates of 87% and 68% at the same time points. Median recurrence-free survival was 10.3 months. The findings, however, were limited by the small sample size (*n* = 35) [36].

Lian et al. [37] conducted a randomised trial comparing adjuvant high-dose interferon (HDI) with toripalimab (anti-PD-1) in patients with completely resected mucosal melanoma. Patients were randomised according to disease stage, tumour site, and PD-L1 expression status. One- and two-year relapse-free survival rates in the HDI and toripalimab arms were 52% versus 52.9% and 25.1% versus 30.5%, respectively. Median recurrence-free survival was 13.9 months in the HDI arm and 13.6 months in the toripalimab arm. No other prospective trials have been reported. However, in a subgroup analysis of 29 patients with mucosal melanoma included in the CheckMate 238 trial, recurrence-free survival was 54% with ipilimumab and 31% with nivolumab, suggesting a non-significant trend favouring ipilimumab in this cohort [38,39]. Across these prospective trials, relapse-free survival rates with immunotherapy are relatively consistent but notably lower than the 65% reported in the CheckMate 915 trial with ipilimumab/nivolumab regimens or monotherapy in cutaneous melanoma [35,38,40].

Very limited data may, however, suggest that certain mucosal melanoma subgroups could derive greater benefit from anti-PD-1 ICIs. In the trial by Lian et al. [37], 50% and 52.1% of patients in the HDI and toripalimab arms, respectively, were found to have PD-1-positive tumours. Among these patients, median recurrence-free survival was 11.1 months with HDI and 17.4 months with toripalimab, indicating a more pronounced benefit of toripalimab in PD-1-positive tumours. The trial was not powered to detect a statistically significant difference in this subgroup. Additionally, in a subgroup of patients with oral mucosal melanoma, retrospective data suggests significant improvement in two-year overall survival and progression-free survival with adjuvant anti-PD-1 combined with chemotherapy compared with chemotherapy alone, consistent with the higher tumour mutational burden and interferon expression seen in head and neck mucosal melanomas compared with other mucosal subgroups [41].

In patients specifically with vulvovaginal melanoma, no prospective trials have assessed the use of immunotherapy in the adjuvant setting, and evidence is limited to retrospective analyses and case series. Wilhite et al. [23] examined the molecular profile of 142 vulvovaginal and 3823 cutaneous melanomas and responses to ICIs. In line with previous evidence, they found a low tumour mutational burden and lower rates of PD-L1 expression in vulvovaginal melanomas compared with cutaneous melanomas, with a corresponding median overall survival of 17.5 months versus 37.9 months in the two groups, respectively, when treated with ICIs. Despite the poorer response, there may still be some benefit, although definitive conclusions are limited by the paucity of evidence. Albert et al. [42] conducted a large retrospective analysis assessing treatment factors and prognosis in 1917 patients with vulval melanoma, of whom 95% underwent surgery and 190 received immunotherapy. In patients with distant metastatic disease, those treated with immunotherapy had a higher two-year overall survival than those who did not (33% versus 12%, *p* = 0.054), although this did not reach statistical significance. This analysis was not stratified by immunotherapy type or disease stage.

Overall, in the context of adjuvant treatment, ICIs have repeatedly demonstrated in prospective phase III trials robust benefits in improving recurrence-free survival in cutaneous melanoma. Comparatively, current evidence in mucosal and particularly vulvovaginal melanoma is extremely limited and suggests a poorer response, likely reflecting their distinct underlying immune and biological characteristics.

#### 3.3.4. Adjuvant Treatment Using Targeted Therapies

In cutaneous melanoma, improved understanding of the high frequency of *BRAF* mutations has led to the development of targeted therapies with *BRAF* and *MEK* inhibitors. In patients with stage III melanoma with *BRAF* V600E or V600K mutations, the phase III COMBI-AD trial demonstrated that adjuvant dabrafenib plus trametinib significantly improved relapse-free survival and overall survival compared with placebo at three and five years of follow-up [43]. There are no prospective trials directly comparing dabrafenib plus trametinib with ICIs, and thus current guidelines recommend either treatment but note higher discontinuation rates in patients treated with dabrafenib plus trametinib compared with ICIs [44].

Unlike cutaneous melanoma, mucosal melanoma shows *BRAF* mutations less frequently (10–15%), and these are more commonly non-V600E/K mutations [23], limiting the applicability of results from the COMBI-AD trial to this group. No studies have specifically evaluated the use of *BRAF*/*MEK* inhibitors in mucosal or vulvovaginal melanoma [39]. A recent systematic review examined the use of *CKIT* inhibitors for unresectable or metastatic mucosal, acral, or chronically sun-damaged melanoma and reported an objective response rate of 14% in patients with mucosal melanoma, with the highest response observed with imatinib (24%) [45]. In a study by Jung et al., patients with mucosal melanoma and confirmed *KIT* mutation demonstrated a response rate of 38% when treated with imatinib. The response rates of *KIT* inhibitors seem to be enhanced in patients with *KIT*-mutant mucosal tumours particularly in exons 11 and 13 with mutations of exon 17 associated with lower progression free survival in multi-variate analysis of prospective and retrospective trials [46].

#### 3.3.5. Adjuvant Treatment Using Chemotherapy

In cutaneous melanoma, adjuvant chemotherapy with alkylating agents was commonly used from the 1970s but achieved poor results, with objective response rates of only up to 15%. With the introduction of *BRAF*/*MEK* targeted therapies and ICIs, multiple systematic reviews have demonstrated significantly improved outcomes compared with conventional chemotherapy. As a result, chemotherapy is not recommended for the adjuvant treatment of high-risk cutaneous melanoma [44,47].

In mucosal melanoma, Lian et al. [48] reported the only randomised prospective controlled trial of adjuvant systemic treatment following resection. The trial included patients who had undergone complete resection of stage II/III disease and were randomised to either high-dose interferon, cisplatin with temozolomide, or observation alone. At a median follow-up of 26.8 months, the median relapse-free survival was statistically significant between the groups: 5.4 months in the observation arm, 9.4 months in the interferon arm, and 20.8 months in the chemotherapy arm. Overall survival also demonstrated statistically significant differences, being 21.2, 40.4, and 48.7 months in the observation, interferon, and chemotherapy groups, respectively.

Extrapolating these findings is challenging, as they contradict current high-quality evidence in cutaneous melanoma, which demonstrates the superiority of immunotherapy over adjuvant chemotherapy, and the trial is limited by small numbers. Consequently, there is insufficient evidence to support a benefit of adjuvant chemotherapy in mucosal melanoma [44].

**Table 2:** Included studies assessing adjuvant treatment in mucosal and vulvovaginal melanoma. Mo—months. Yr—years.

Author	Trial Design	Patient Group	N	Intervention	Recurrence-Free Survival (95% CI)	Median RFS (95% CI)
Mucosal Melanoma						
Kottschade et al. [36]	Single-arm, multicentre	Mucosal melanoma (R0/R1 resection)	35	Flip-dose ipilimumab + nivolumab, then maintenance nivolumab (1 year total)	1 yr: 50% (31–66%); 2 yr: 37% (19–55%)	10.3 mo (5.7–25.8)
Lian et al. [37]	Randomised controlled trial	Mucosal melanoma (R0/R1 resection)	145	High-dose interferon (HDI) vs. toripalimab for 1 year or until recurrence/toxicity/withdrawal	1 yr: HDI 52% (38.6–63.8%); toripalimab 52.9% (40.3–63.9%) 2 yr: HDI 25.1% (14.8–36.8%); toripalimab 30.5% (19.7–41.9%)	HDI 13.9 mo (8.3–19.0); toripalimab 13.6 mo (8.3–19.0)
Wu et al. [41]	Single-centre, retrospective cohort	Oral mucosal melanoma (complete resection)	193	Dacarbazine/cisplatin + HDI vs. dacarbazine/cisplatin + ICI	—	Chemo 10.9 mo; HDI 43.6 mo; PD-1 53.6 mo
Lian et al. [48]	Randomised controlled trial	Stage II/III mucosal melanoma (complete resection)	189	Observation vs. HDI vs. temozolomide/cisplatin	—	Obs. 5.4 mo (4.2–6.6); HDI 9.4 mo (7.9–10.9); Tem/Cis 20.8 mo (17.9–23.7)
Vulvovaginal Melanoma						
Wilhite et al. [23]	In vitro, retrospective cohort	Tissue samples (3965 total; 142 vulvovaginal)	3965	ICIs	—	Vulvovaginal: 17.5 mo; Cutaneous: 37.9 mo
Albert et al. [42]	Retrospective cohort	Vulval melanoma	1917	Immunotherapy (unspecified)	—	—

#### 3.3.6. Non-Resectable or Metastatic Disease

The introduction of targeted therapy and ICIs has dramatically improved outcomes for patients with advanced, unresectable cutaneous melanoma, achieving durable disease control and potentially a cure in approximately 50% of patients [1,4]. However, as in the adjuvant setting, the benefits of immunotherapy are less pronounced in mucosal, and particularly vulvovaginal, melanoma with only retrospective studies in the mucosal melanoma and vulvovaginal groups. For advanced and non-resectable *BRAF*-mutant cutaneous melanoma, combination *BRAF*/*MEK* inhibitors produce high initial response rates and at least a partial objective response in most patients, with only mild toxicity. However, despite these high initial response rates, around 50% of patients develop resistance within one year and 80% within five years [49,50].

ICIs also demonstrate efficacy for patients with both *BRAF*-mutant and wild-type advanced cutaneous melanoma. The anti-PD-1 antibodies pembrolizumab and nivolumab have shown superior efficacy to ipilimumab, with higher response rates and improved five-year progression-free and overall survival. Combination therapy with ipilimumab and nivolumab has demonstrated the highest response rates and five-year overall and progression-free survival, but is associated with more frequent toxicity [51,52].

Given the proven efficacy of both targeted therapy and ICIs, studies have been conducted to assess outcomes based on the sequence of their use. Randomised prospective trial data support the use of combination ICIs as the first-line treatment for advanced non-resectable cutaneous melanoma, showing improvements in overall survival and progression-free survival compared with patients treated initially with *BRAF*/*MEK* inhibitors [53,54]. The phase III DREAMseq trial randomised patients with advanced, non-resectable cutaneous melanoma to Arm A (nivolumab plus ipilimumab until progression, followed by dabrafenib plus trametinib) or Arm B (dabrafenib plus trametinib until progression, followed by nivolumab plus ipilimumab). Two-year overall survival was 71.8% for Arm A compared with 51.5% for Arm B. Objective response rates were 46% in Arm A and 43% in Arm B [54].

For metastatic or unresectable mucosal melanoma, prospective evidence remains limited, with most studies comprising small observational cohorts or retrospective analyses (included studies summarised in Table 3). Moya-Plana et al. [55] conducted a single-centre prospective cohort study of patients with unresectable locally advanced and/or metastatic mucosal melanoma treated with either ipilimumab or pembrolizumab. Forty-four patients were enrolled, including 14 with vulvovaginal primaries. The objective response rate was 20% overall, 8% for ipilimumab, and 35% for pembrolizumab, with no significant difference observed according to tumour location. Two-year overall survival was 34% with ipilimumab and 44% with pembrolizumab, while median overall survival was 12 months and 16.2 months, respectively. A post hoc analysis of the KEYNOTE-001, KEYNOTE-002, and KEYNOTE-006 trials evaluated pembrolizumab in metastatic melanoma and reported an objective response rate of 19% in mucosal melanoma, compared with 33% in cutaneous melanoma, with a median overall survival of 11.3 months [56].

As in cutaneous melanoma, patients with unresectable mucosal melanoma may benefit more significantly from combination therapy with ipilimumab and nivolumab than from checkpoint inhibitor monotherapy. In a pooled post hoc analysis of phase III trials, D’Angelo et al. [57] assessed 86 patients with unresectable mucosal melanoma treated with nivolumab monotherapy and 35 treated with combined nivolumab and ipilimumab. Median progression-free survival was 3.0 months with nivolumab, 5.9 months with combination therapy, and 2.7 months with ipilimumab. Objective response rates were 23.3%, 37.1%, and 8.3% for nivolumab, combination therapy, and ipilimumab, respectively. This effect was more pronounced in patients with PD-L1 expression greater than 5%, with objective response rates of 53.3%, 60%, and 14.3% for nivolumab, combination therapy, and ipilimumab, respectively. The difference in objective response rates between patients with PD-L1 expression above and below 5% was greater in mucosal melanoma than in cutaneous melanoma.

Similar findings were reported in a large multicentre retrospective cohort study comparing anti-PD-1 monotherapy with combination immunotherapy in 545 patients with advanced, unresectable, or metastatic mucosal melanoma. Objective response rates were 29% with anti-PD-1 monotherapy and 31% with combination therapy. Median progression-free survival was 5 months and 4 months in the anti-PD-1 monotherapy and combination therapy groups, respectively. Median overall survival was 19 months and 21 months, and three-year overall survival rates were 33% and 30%, respectively, for anti-PD-1 monotherapy and combination therapy. Overall survival was initially numerically, but not statistically, higher with combination therapy; however, the survival curves crossed at the two-year mark [58]. In the above two studies, no differences in objective response rates were observed across the primary sites of mucosal melanoma.

There are no prospective studies assessing the use of ICIs in vulvovaginal melanoma and studies looking specifically at the use of immunotherapy in vulvovaginal melanoma are limited to small, single centre retrospective cohort studies and case series. In a retrospective cohort study, data from 13 patients with unresectable or metastatic vulvovaginal melanoma were combined with a further 10 patients identified from previously published cases in the literature. The overall response rate to ICIs was 30.4%, with a median progression-free survival of 4.0 months and a median overall survival of 17 months. Ipilimumab was again demonstrated to be inferior to PD-1 monotherapy or combination immunotherapy [59]. Another small retrospective case series reported similar response rates of 28.5% to immune checkpoint therapy, with patients receiving anti-PD-1 monotherapy achieving longer progression-free survival than those treated with anti-CTLA-4 therapy [60]. These limited findings are consistent with response and survival rates reported in larger analyses of mucosal melanoma. Although there is a lack of prospective, randomised evidence and the available trials include patients with varied baseline characteristics, the evidence to date supports a possible role for immune checkpoint inhibitor therapy in the treatment of advanced, metastatic, unresectable melanoma. The benefit does not appear to be as marked as in cutaneous melanoma, likely due to underlying differences in tumour biology and immune profile. Patient counselling in a supported environment is recommended to facilitate informed, patient-centred decision-making. This should include discussion of potential serious side effects and the associated risk of reduced quality of life, alongside any potential improvements in outcome.

## 4. Future Directions

This review highlights the significant paucity of evidence underpinning the diagnosis and management of mucosal and vulvovaginal melanomas which is reflected in the stagnation of survival outcomes. In cutaneous melanoma, ongoing studies are identifying new biomarkers to assist in the earlier diagnosis and assessment of metastatic potential which, if successful, will likely further broaden the gap between advances in cutaneous and mucosal melanoma subtypes. Emerging evidence suggests that profiling fatty acids and protein signatures in small extracellular vesicles may provide a liquid biopsy approach for earlier cutaneous melanoma detection and stage specific disease monitoring. Similar approaches are being investigated in breast and prostate cancers [61].

There has been some progress made in identifying the key biological and genetic differences between cutaneous, mucosal and vulvovaginal melanoma, thus aiding our understanding of the poorer response and outcomes of these patients in response to current therapies. The key challenges limiting progress in mucosal and vulvovaginal melanomas currently include delayed diagnosis resulting in advanced disease, the lack of therapeutic gene and protein targets and the difficulty in designing robust studies given the rarity of these conditions. Future key areas of research must include more sensitive and specific diagnostic tools which facilitate earlier detection, as achieved in the context of cutaneous melanoma. Additionally, with acknowledgement of the distinct genetic and immunogenic features, identification of novel and more disease specific therapeutic targets will provide the first steps in formulation of more efficacious treatment options.

The largest overarching challenge in both mucosal and vulvovaginal melanoma is the rarity of these conditions, which limits potential recruitment for larger studies. Progress in this area will depend on collaborative efforts including the formation of international patient registries to aid in the identification of larger numbers of patients, data collection and international collaborative studies. Tissue banking also provides the possibility of in vitro or translation studies, which may be particularly helpful in further identification of future therapeutic targets. Additionally, with the move towards targeted therapy and innovative trial designs, such as basket trials with a novel approach of grouping tumours by molecular and genetic mutations rather than histopathological origin, may also provide meaningful therapeutic insights. This would be particularly useful in assessing the utility of future therapeutic strategies, given that large scale randomised controlled trials are not likely to be feasible for such a rare condition.

## 5. Conclusions

Compared with cutaneous melanoma, mucosal melanomas—and vulvovaginal melanomas in particular—remain poorly understood, with their rarity limiting the feasibility of large, high-powered studies. While advances in the understanding and treatment of cutaneous melanoma have translated into clear improvements in overall survival across all disease stages, knowledge of the pathogenesis, molecular mechanisms, and immune profile of vulvovaginal melanoma remains limited, with little evidence to support the benefit of surgery or ICIs. As a result, recurrence rates and overall survival outcomes in this population remain poor, with marked heterogeneity in treatment approaches. This review clearly compares and contrasts features of cutaneous melanoma, mucosal melanoma and vulvovaginal melanoma to reinforce the need to consider these tumour types as three distinct, albeit related pathologies. The synthesis of current evidence allows for a better understanding of these poorer outcomes and the underlying biological reasons for this as well as identifying key directions forward. A deeper understanding of the unique biology of mucosal and vulvovaginal melanomas is urgently needed to inform more effective therapeutic strategies.

## Figures and Tables

**Table 1:** Main characteristics of different melanoma types.

	Type of Melanoma		
Characteristics	Cutaneous	Mucosal	Vulvovaginal
Age	40–60	60–80	60–70
Site	trunk	head and neck	vulva
	limbs	anorectal	vaginal walls
	face		
Affected cells	epidermal melanocytes (skin)	mucosal melanocytes (nasal, GI, genital)	mucosal melanocytes (vulva/vagina)
UVR DNA damage	strongly associated	n/a	n/a
Pigmentation	present	frequently amelanotic	present (vulval lesions) often amelanotic (vagina)
Presentation	changing mole (de novo) ABCDE criteria bleeding itching	nasal obstruction/bleeding rectal bleeding/discharge dysphagia	vulval/vaginal mass bleeding discharge
Growth pattern	radial and vertical	deep/invasive	deep/invasive
LN involvement	variable incidence	high incidence	high incidence
Stage at diagnosis	localised (early detection)	delayed diagnosis	advanced stage
Metastasis pattern	later metastasis (liver, lungs, bones, brain)	early metastasis/aggressive	often presented with local or distant metastasis
Mutational burden	high	low	low
Immune response	effective	reduced/variable	reduced/variable
Prognosis	good	poor	poor

UVR: ultraviolent radiation; n/a: not applicable; ABCDE: Asymmetry, Border irregularity, Colour variation, Diameter > 6 mm, Evolving; LN: lymph node.

**Table 3:** Included studies assessing systemic treatments for unresectable or metastatic mucosal and vulvovaginal melanoma. Mo—months, ipi—ipilimumab, pembro—pembrolizumab, nivo—nivolumab.

Author	Trial Design	Patient Group	N	Intervention	Progression-Free Survival (95% CI)	Objective Response Rate	Overall Survival (95% CI)
Mucosal Melanoma							
Moya Plana et al. [55]	Prospective cohort	Unresectable or metastatic mucosal melanoma	44	Ipilimumab or pembrolizumab	Ipi:3 mo (2.5–4.6) Pembro: 5 mo (2.6–33.1)	Ipi 8.2% Pembro 35%	1 yr: Ipi 54% (32.2–71.2), Pembro 57% (31.5–75.6) 2 yr: Ipi 34% (16.0–53.9), Pembro 44% (20.8–65.2)
Hamid et al. [56]	Post hoc analysis of KEYNOTE-001, 002 & 006	Unresectable or metastatic mucosal melanoma	84	Pembrolizumab	2.8 mo (2.7–2.8)	19% (95% CI 11–29)	11.3 mo (7.7–16.6)
D’Angelo et al. [57]	Subgroup analysis of Phase I and III trials	Unresectable/metastatic mucosal melanoma, cutaneous melanoma	1417 (157 mucosal)	Nivolumab vs. ipilimumab vs. combination	Mucosal: Nivo 3.0 mo (2.2–5.4) Ipi 2.7 mo (2.6–2.8) Combination 5.9 mo (2.2–NR)	Mucosal: Nivo 23.3% (14.8–33.6) Ipi 8.3% (1.8–22.5) Combination\ 37.1% (21.5–55.1)	Not reported
Dimitriou et al. [58]	Multicentre, retrospective cohort	Unresectable or metastatic mucosal melanoma	545	Anti-PD-1 vs. Anti-PD-1 + ipilimumab	Anti-PD-1: 5 mo (4–6) Combination: 4 mo (3–6)	Anti-PD-1 29% (24–34) Combination 31% (25–38)	Anti-PD-1 19 mo (16–24) Combination 21 mo (19–27)
Vulvovaginal Melanoma							
Wohlmuth et al. [59]	Single-centre, retrospective cohort	Unresectable or metastatic vulvovaginal melanoma	23	Immune checkpoint inhibitors	4.0 mo (2.7–5.3)	30.4% (11.6–49.2)	17.0 mo (12.7–21.3)
Indini et al. [60]	Single-centre, prospective cohort	Metastatic vulvovaginal/cervical melanoma	7	Immune checkpoint inhibitors	—	28.5%	—

## Data Availability

No new data were created or analyzed in this study. Data sharing is not applicable to this article.

## Abbreviations

ICIs	immune checkpoint inhibitors
CTLA-4	cytotoxic T-lymphocyte associated protein 4
PD-1	programmed cell death protein 1
PD-L1	programmed death-ligand 1
UVR	ultraviolet radiation
MAPK	mitogen-activated protein kinase
NK cells	natural killer cells
HDI	high-dose interferon
